# Chronic Obstructive Pulmonary Disease Is Not Associated with *KRAS* Mutations in Non-Small Cell Lung Cancer

**DOI:** 10.1371/journal.pone.0152317

**Published:** 2016-03-23

**Authors:** Ali Saber, Anthonie J. van der Wekken, Gerald S. M. A. Kerner, Maarten van den Berge, Wim Timens, Ed Schuuring, Arja ter Elst, Anke van den Berg, T. Jeroen N. Hiltermann, Harry J. M. Groen

**Affiliations:** 1 Department of Pathology and Medical Biology, University Medical Center Groningen, University of Groningen, Groningen, the Netherlands; 2 Department of Pulmonary Diseases, University Medical Center Groningen, University of Groningen, Groningen, the Netherlands; Comprehensive Pneumology Center, GERMANY

## Abstract

Mutations in epithelial growth factor receptor *(EGFR)*, as well as in the EGFR downstream target *KRAS* are frequently observed in non-small cell lung cancer (NSCLC). Chronic obstructive pulmonary disease (COPD), an independent risk factor for developing NSCLC, is associated with an increased activation of EGFR. In this study we determined presence of *EGFR* and *KRAS* hotspot mutations in 325 consecutive NSCLC patients subjected to *EGFR* and *KRAS* mutation analysis in the diagnostic setting and for whom the pulmonary function has been determined at time of NSCLC diagnosis. Information about age at diagnosis, sex, smoking status, forced vital capacity (FVC) and forced expiratory volume in 1 sec (FEV_1_) was collected. Chronic obstructive pulmonary disease(COPD) was defined according to 2013 GOLD criteria. Chi-Square, student t-test and multivariate logistic regression were used to analyze the data. A total of 325 NSCLC patients were included, 193 with COPD and 132 without COPD. COPD was not associated with presence of *KRAS* hotspot mutations, while *EGFR* mutations were significantly higher in non-COPD NSCLC patients. Both female gender (HR 2.61; 95% CI: 1.56–4.39; p<0.001) and smoking (HR 4.10; 95% CI: 1.14–14.79; p = 0.03) were associated with *KRAS* mutational status. In contrast, only smoking (HR 0.11; 95% CI: 0.04–0.32; p<0.001) was inversely associated with *EGFR* mutational status. Smoking related G>T and G>C transversions were significantly more frequent in females (86.2%) than in males (61.5%) (p = 0.008). The exon 19del mutation was more frequent in non-smokers (90%) compared to current or past smokers (36.8%). In conclusion, *KRAS* mutations are more common in females and smokers, but are not associated with COPD-status in NSCLC patients. *EGFR* mutations are more common in non-smoking NSCLC patients.

## Introduction

Chronic obstructive pulmonary disease (COPD) is associated with lung cancer also after accounting for other respiratory diseases and smoking [1–2]. An increased risk of lung cancer in COPD patients was evident in a meta-analysis [2]. About one third of smokers with COPD died of lung cancer within a follow-up of 14.5 years [3]. On the other hand, 50–70% of the lung cancer patients have COPD according to results of pulmonary function tests at time of diagnosis [4]. In a more recent, large prospective study, the association between COPD and lung cancer was largely explained by smoking [5]. The odds ratio (OR) for patients diagnosed with COPD to develop lung cancer within a period of 6 months was 11.4. However, the OR dropped to 6.8 after correction for smoking [5]. This is consistent with the notion that COPD has been recognized as an independent risk factor for developing lung cancer [6].

KRAS is involved in regulation of cell proliferation [7]. Mutations in *KRAS* are mostly found in codons 12, 13 and 61 and result in constitutive activation of the protein [8]. *KRAS* mutations are observed more frequent in smoking patients with adenocarcinoma (5–40%) than in the other subtypes of lung cancer [7, 9]. Mutations in *KRAS* are associated with poorer prognosis of NSCLC patients [10]. Moreover, a COPD-like airway inflammation can increase lung carcinogenesis in the presence of the p.G12D *K-ras* activating mutation in a mouse model [11].

EGFR plays a crucial role in wound healing and tissue repair in the lung, especially in the bronchial wall. Overexpression of EGFR was reported in the bronchial mucosa of non-smoking asthmatic individuals compared to normal controls [12]. Moreover, prolonged activation of EGFR leads to metaplasia [13]. Exposure of epithelial cells to cigarette smoke induced aberrant phosphorylation and activation of EGFR and this may subsequently mediate development of lung cancer [14–15]. Mutations in the kinase domain also lead to activation of the EGFR pathway independent of binding to its ligand [16]. These activating *EGFR* mutations are common in non-small cell lung cancer (NSCLC) with a frequency of about 10–15% in Caucasians [17–18]. *EGFR* mutations have been associated with non-smoking NSCLC patients [19]. The p.(L858R) in exon 21 (referred to as L858R) and deletions in exon 19 (referred to as exon 19del) of the *EGFR* gene are the most commonly observed activating mutations [20]. We previously showed a significant association between *EGFR* mutations and clinical outcome [21]. *In vivo* studies in mouse models conditionally expressing either the L858R or an exon19del mutant allele of the human *EGFR* gene have supported the role of these mutations in initiation and development of lung cancer [22].

Smoking is a known risk factor for both COPD and lung cancer [23–24]. *KRAS* mutations are described as a signature for cigarette smoking [25], while *EGFR* mutations are more common in non-smokers. We hypothesize that *KRAS* mutations are positively associated with COPD status in NSCLC patients, while activating *EGFR* mutations are negatively associated with COPD in NSCLC patients. To study this hypothesis we analyzed NSCLC patients screened for the presence of *EGFR* and *KRAS* mutations in a diagnostic setting and investigated whether the presence of *EGFR* and *KRAS* mutations in NSCLC patients was related to COPD.

## Materials and Methods

### Patients

Consecutive patients with advanced NSCLC, diagnosed between November 2008 and July 2012, and for whom *KRAS* and *EGFR* mutation analysis was performed in a clinical setting, were selected for this study. In this cohort we further selected patients for whom lung function data were available. All patients had stage IV NSCLC and had one or more visceral metastases at diagnosis. Previously, 165 of the NSCLC patients have been described in a study on *EGFR* and *KRAS* mutations in relation to clinical outcome [21]. Patients with NSCLC post lung transplantation were excluded from this study. For all patients, data on gender, smoking status (including pack year if available), age at diagnosis, stage at diagnosis according to the 6^th^ TNM edition, localization of metastases, start date and (different) lines of treatment were collected. Data on lung function was newly collected for all patients included in this study. All procedures and protocols were performed according to the guidelines for good clinical practice and after informed consent was obtained from all patients.

### Informed Consent and Ethics

Written informed consent for blood and tumor tissue from all patients was obtained before biobanking. This procedure was approved by the Medical Ethical Committee of the University Medical Center Groningen. This study was conducted in accordance with the provisions of the Declaration of Helsinki and Good Clinical Practice guidelines. For this study, all patient data were anonymized and de-identified prior to analysis. Besides the mutational analysis, pulmonary function tests were performed as part of routine diagnostic approach and the outcome of these tests was documented in the patient file and communicated with patients. Due to the retrospective nature of this study, under Dutch Law for human medical research (WMO), no specific permission was compulsory from the Institutional Review Board.

### Pulmonary function testing

Spirometry was performed with a daily-calibrated pneumotachograph (MasterscreenPneumo, Jaeger, Wurzburg, Germany) according to standardized guidelines [26]. Lung function tests are provided after bronchodilatator (salbutamol 100 microgram). Patients were defined as having COPD if the forced expiratory volume in 1 sec (FEV_1_)/forced vital capacity (FVC) (FEV1/FVC) was <0.70 with fixed bronchial obstruction over time not due to endobronchial tumor obstruction. Staging of COPD was performed according to GOLD criteria [27].

### Histology and *KRAS/EGFR* molecular testing

Tumor samples were obtained either by bronchoscopy, transthoracic lung biopsies and/or from pulmonary resections. Histological subtyping was performed according to 2004 WHO criteria [28]. Mutational analysis was performed as previously described [21].

### Statistics

For normally distributed data we show mean and standard deviation (SD) and used a student t-test to determine significant differences. For not normally distributed data median and range are given and Chi-Square test is used to determine significance. Logistic regression was performed to study whether the presence of COPD had any effect on *KRAS* or *EGFR* mutational status using sex, age, histology, and smoking as covariates. Statistical analysis was performed using SPSS version 22.0. Nominal *P*-values less than 0.05 were considered significant. Data are available in S1 Table.

## Results

### Patient characteristics and *KRAS*/*EGFR* mutations

A total of 325 stage IV NSCLC patients were included. Over 80% had adenocarcinoma, 174 (53.5%) were male and 151 (46.5%) female. The mean age at time of diagnosis was 63.6 (±10.5 years). One hundred and five patients (32.3%) had a *KRAS* mutation. For 1 out of 105 patients with a *KRAS* mutation, the type of mutation was inconclusive with a positive high resolution melting (HRM) PCR result, but with a wild type sequence based on the Sanger sequencing result. For one patient with an *EGFR* mutation, the *KRAS* mutation status was not available. The remaining 219 patients did not have mutations in the *KRAS* hotspot region. Twenty-nine patients (8.9%) had an *EGFR* mutation. In five patients, the *EGFR* mutational status was not available; four of these patients did have a *KRAS* mutation. The other 291 patients did not have *EGFR* mutations in the hotspot regions. The mean age of the males was higher than the mean age of the females (66.3 ±9.8 years vs. 60.5 ±10.5 years; p<0.001). Males showed a significant higher number of smoking pack years than females (mean 37.5 ±20.6 pack years vs. 30.1 ±15.7 pack years; p = 0.015).

### Patient characteristics in COPD stratified subgroups

Almost 60% (193/325) of the NSCLC patients had COPD. Two third of the COPD cases were males. The distribution of females was almost equal in COPD and non-COPD groups (Table 1). Mean age in the COPD group was higher with 65.6 years (±9.9 years) compared to the non-COPD group with 60.6 (±10.8 years) (p<0.001). We found a significant relationship between smoking and COPD, 62.6% of current or past smokers had COPD, while only 18.2% of non-smokers had COPD (p<0.001). A logistic regression model for COPD using sex, age and smoking as covariates revealed significant associations with age (Hazard ratio [HR] 1.05; 95% confidence interval [CI]: 1.02–1.07; p<0.001) and smoking (HR 8.28; 95% CI: 2.61–26.24; p<0.001), but not with gender (Table 2).

**Table 1:** NSCLC patient characteristics according to COPD status.

Characteristics	COPD (%) N = 193	Non-COPD (%) N = 132	p-value
Sex			
Female	77 (51)	74 (49)	0.004†
Male	116 (66.7)	58 (33.3)	
Age at diagnosis, Mean (±SD)	65.6 (±9.9)	60.6 (±10.8)	<0.001*
Histology			
Adenocarcinoma	154 (59)	107 (41)	0.777†
Adeno-squamous	14 (66.7)	7 (33.3)	
NSCLC-NOS	25 (58.1)	18 (41.9)	
Smoking status^#^			
Current or past smoker	184 (62.6)	110 (37.4)	<0.001†
Non-smoker	4 (18.2)	18 (81.8)	
FEV1%, Mean (±SD)	72.8 (±18.7)	90.2 (±19.4)	-
FEV1/FVC ratio, Mean (±SD)	0.58 (±0.09)	0.76 (±0.05)	-
*KRAS* mutation^#^			
Yes	66 (62.9)	39 (37.1)	0.404†
No	127 (58)	92 (42)	
*EGFR* activating mutation^#^			
Yes	9 (31)	20 (69)	0.001†
No	180 (61.9%)	111 (38.1)	

* T-test;

^†^Chi-square test;

^#^Missing data for smoking (n = 9), KRAS (n = 1) and EGFR (n = 5) status.

**Table 2:** Logistic regression analysis of patient characteristics associated with COPD, *KRAS* and *EGFR*.

Characteristics	COPD			KRAS			EGFR		
	HR	95% CI	p-value	HR	95% CI	p-value	HR	95% CI	p-value
Sex	0.78	0.48–1.29	0.33	2.61	1.56–4.39	<0.001	1.21	0.51–2.90	0.66
Age	1.05	1.02–1.07	<0.001	0.99	0.97–1.01	0.39	0.98	0.95–1.02	0.39
Smoking	8.28	2.61–26.24	<0.001	4.10	1.14–14.79	0.03	0.11	0.04–0.32	<0.001
COPD	-	-	-	1.29	0.77–2.18	0.34	0.44	0.18–1.09	0.08

HR: Hazard ratio; CI: Confidence interval

### COPD and tumor *KRAS/EGFR* hotspot mutations

**Fig 1:**
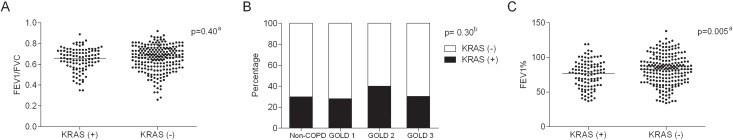
*KRAS* mutations and severity of airflow obstruction. (A) FEV1/FVC, (B) COPD GOLD classification and (C) FEV1 percentage in *KRAS* mutant and wildtype patients with NSCLC. ^a^P-value was calculated by student t-test. ^b^P-value was calculated by Chi-square test.

*KRAS* mutations were observed more often in females compared to males (65/151 (43%) versus 40/173, (23%); p<0.001) and also more often in current or past smokers (34.5%) than in non-smokers (13.6%) (p = 0.045) (Table 3). *KRAS* mutations were not significantly different between COPD (34.2%) and non-COPD patients (29.8%) (Table 1), sothe presence of *KRAS* mutations were independent of COPD. Stratification according to FEV1/FVC and GOLD stage did not reveal a significant association with presence of *KRAS* mutation (Fig 1A and 1B). However, FEV1 percentage as a continuous variable was significantly related to the presence of *KRAS* mutations (Fig 1C), but not in a multivariate analysis. Putting the variables (sex, age, smoking and COPD) in a logistic regression model confirmed the significant association between *KRAS* hotspot mutations with female sex (HR 2.61; 95% CI: 1.56–4.39; p<0.001) and smoking (HR 4.10; 95% CI: 1.14–14.79; p = 0.03) (Table 2).

**Table 3:** NSCLC patient characteristics and*KRAS/EGFR* mutation*.

Characteristics	*KRAS* mutation		Pearson Chi-Square	*EGFR* mutation		Pearson Chi-Square
	No (%)	Yes (%)		No (%)	Yes (%)	
Sex						
Female	86 (57)	65 (43)	<0.001	130 (87.8)	18 (12.2)	0.07
Male	133 (76.9)	40 (23.1)		161 (93.6)	11 (6.4)	
Smoking status						
Current or past smoker	192 (65.5)	101 (34.5)	0.045	270 (93.4)	19 (6.6)	<0.001
Nonsmoker	19 (86.4)	3 (13.6)		12 (54.5)	10 (45.5)	

* Missing data for smoking (n = 9), KRAS (n = 1) and EGFR (n = 5) status.

*EGFR* mutations showed a trend to a higher frequency in females compared to males, (p = 0.073). Ten out of 22 (45.5%) non-smokers had activating *EGFR* mutations, while only 19 out of 289 (6.5%) of the current or past smokers had an *EGFR* mutation (p<0.001). *EGFR* mutations were observed more often in the non-COPD (20/131, i.e. 15.3%) as compared to the COPD group (9/189, i.e. 4.8%)(p = 0.001) (Table 3). Using sex, age, smoking and COPD as covariates in a logistic regression model for *EGFR* mutations, confirmedsignificant inverse association between smoking (HR 0.11; 95% CI: 0.04–0.32; p<0.001) and *EGFR* mutational status (Table 2).

### COPD and type of *KRAS/EGFR* mutations

The *KRAS* p.(G12C) was the most common amino acid change in both male and female with a frequency of approximately 41%. The p.(G12V) and p.(G12D) mutations were the second most frequent mutations in females and males, with a frequency of 20% and 25.6%, respectively (Table 4). Forty-three percent of the *KRAS* mutations in the current or past smoker group were p.(G12C) mutations, while none of the non-smoking patients had this mutation. In addition, G>T and G>C transversions in *KRAS* occurred in 86.2% of the females and in 61.5% of the males. The G>A transition was more common in males than in females (p = 0.008)(Table 5). COPD status was not associated with any type of *KRAS* amino acid changes or nucleotide substitutions.

**Table 4:** Distribution of different *KRAS* amino acid changes in advanced NSCLC patients*.

Characteristics	*KRAS* amino acid change					Pearson Chi-Square
	p.(G12C) (%)	p.(G12V) (%)	p.(G12A) (%)	p.(G12D) (%)	Other (%)	
Sex						
Female	27 (41.5)	13 (20)	7 (10.8)	6 (9.2)	12 (18.8)	0.20
Male	16 (41)	5 (12.8)	2 (5.1)	10 (25.6)	6 (15.4)	
Histology						
Adenocarcinoma	41 (42.7)	16 (16.7)	8 (8.3)	13 (13.5)	18 (18.8)	0.26
NSCLC NOS	2 (25)	2 (25)	1(12.5)	3 (37.5)	0	
Smoking status						
Current or past smoker	43 (43)	17 (17)	7 (7)	15 (15)	18 (18)	0.24
Nonsmoker	0	1 (33.3)	1 (33.3)	1 (33.3)	0	
COPD						
Yes	27 (40.9)	11 (16.7)	6 (9.1)	11 (16.7)	11 (16.7)	0.99
No	16 (42.1)	7 (18.4)	3 (7.9)	5 (13.2)	7 (18.4)	

* Missing data for smoking (n = 1) status and type of *KRAS* mutation (n = 1); p.(G12C) and p.(G12V) (G>T), p.(G12A) (G>C), p.(G12D) (G>A).

**Table 5:** Distribution of different *KRAS* nucleotide changes in advanced NSCLC patients*.

Characteristics	*KRAS* mutations			Pearson Chi-Square*
	Transversions G>T, G>C (%)	Transitions G>A (%)	Other (%)	
Sex				
Female	56 (86.2)	7 (10.8)	2 (3.1)	0.008
Male	24 (61.5)	14 (35.9)	1 (2.6)	
Histology				
Adenocarcinoma	75 (78.1)	18 (18.8)	3 (3.1)	0.41
NSCLC NOS	5 (62.5)	3 (37.5)	0	
Smoking				
Current or past smoker	77 (77)	20 (20)	3 (3)	0.83
Nonsmoker	2 (66.7)	1 (33.3)	0	
COPD				
Yes	50 (75.8)	14 (21.2)	2 (3)	0.93
No	30 (78.9)	7 (18.4)	1 (2.6)	

* Missing data for smoking (n = 1) status and type of *KRAS* mutation (n = 1).

Of all *EGFR* mutation positive cases the percentage of patients with an exon 19del was not significantly different between females (11/18) and males (5/11). In non-smokers, 9 out of 10 *EGFR* mutation positive cases had an exon 19del (Table 6), whereas in current or past smokers only 7 out of 19 patients with an *EGFR* mutation had an exon 19del.

**Table 6:** Distribution of different *EGFR* mutations in advanced NSCLC patients.

Characteristics	*EGFR* mutations			Pearson Chi-Square
	Exon 19del (%)*	p.(L858R) (%)†	Other (%)‡	
Sex				
Female	11 (61.1)	4 (22.2)	3 (16.7)	0.69
Male	5 (45.5)	3 (27.3)	3 (27.3)	
Histology				
Adenocarcinoma	16 (57.1)	6 (21.4)	6 (21.4)	0.20
NSCLC NOS	0	1 (100)	0	
Smoking				
Current or past smoker	7 (36.8)	6 (31.6)	6 (31.6)	0.02
Nonsmoker	9 (90)	1 (10)	0	
COPD				
Yes	5 (55.6)	3 (33.3)	1 (11.1)	0.60
No	11 (55)	4 (20)	5 (25)	

* One patient had an exon 19del and p.(T790M),

^†^One patient had a p.(L858R) and p.(T790M),

^‡^ One patient had a p.(G719S) and p.(S768I) and another patient had a p.(G719C) and an exon 20 insertion.

## Discussion

In contrast to our hypothesis we showed that COPD is not associated with the presence of *KRAS* mutations in lung cancer, whereas presence of *EGFR* mutations was more frequent in non-COPD as compared to COPD lung cancer patients, after correcting for sex and smoking. We found significantly higher mean age in the COPD group as compared to the non-COPD group. This finding is consistent with the fact that the COPD prevalence increases with age [29].

*KRAS* mutations were identified in 32% of the NSCLC patients, which mainly included adenocarcinoma patients. We observed a relationship between presence of *KRAS* hotspot mutations and smoking status consistent with previous studies [25, 30], but not with COPD. In other studies no relation between smoking and the presence of *KRAS*mutations have been observed in lung cancer patients[25, 30–32]. These differences may be caused by differences in selecting study groups, ethnicity, number of patients and smoking status. The lack of an association with COPD is in concordance with the results reported ina recent study [33].

Although smoking females were younger and lighter smokers based on pack years than the males, we noticed that *KRAS* mutations were more common in smoking females than in smoking males with NSCLC. This supports an increased susceptibility of females to cigarette carcinogens as reported previously [34]. Moreover, these results are also consistent with a previous study showing that females had a higher OR for lung cancer at every level of tobacco exposure [35]. This elevated vulnerability to smoking may be caused by the higher expression levels of genes encoding tobacco carcinogen-metabolizing enzymes, such as CYP1A1 and CYP1B1, in normal lung tissue of female smokers in comparison to male smokers [36]. Uppstad and colleagues [37] also showed higher expression of CYP1A1 in cell lines derived from lung adenocarcinoma of female compared to cell lines derived from adenocarcinomas of male patients.

Although we observed the smoking related p.(G12C) *KRAS* mutation at the same frequency in both genders, smoking related transversions, i.e. G>T and G>C, were significantly more common in females than in males. In a previous study with a sample size of over 2,500 patients, the c.34G>T; p.(G12C) *KRAS* mutation occurred more frequent in females and current or past smokers, while the c.35G>A; p.(G12D) *KRAS* mutations were more frequent in never smokers [20]. This suggests again that females are more susceptible of cigarette smoke related *KRAS* mutations compared to males.

We showed that *EGFR* activating mutations were more common in females, non-smokers and in non-COPD NSCLC patients. In a recent study, *EGFR* mutations were seen in 12.8% (51/399) of lung cancer patients without COPD and in 6.3% (7/111) of patients with COPD [38]. Suzukiand colleagues [39]identified *EGFR* mutations in 32% (56/177) of the non-COPD and in 8% (4/52) of the COPD NSCLC patients. Lim and colleagues[33] found *EGFR* mutations in 37.3% (91/244) of non-COPD and in 16% (17/106) of COPD patients. They also found an inverse association between the presence of *EGFR* mutation with severity of airflow obstruction. The finding that *EGFR* mutations are more common in non-COPD lung cancer patients might indicate that lung cancer development is dependent on activating *EGFR* mutations in non-COPD patients.Chronic pulmonary diseases, such as severe asthma and COPD, cause an increased activation of the epithelial growth factor receptor (EGFR)[12, 40]. Moreover, COPD is characterized by epithelial inflammatory reactions and many pro-inflammatory chemokines and growth factors are induced by transcription factor Nuclear Factor kB (NFkB). This transcription factor can be activated via physical and chemical stress such as smoke [41]. In addition, increased activation of EGFR by oxidative stress, which is involved in pathogenesis of COPD, or cigarette smoke can occur in human bronchial epithelial cells [14–15, 42]. All together suggesting that EGFR activation in COPD is induced by smoking, oxidative stress and subsequently by inflammation probably via NFkB.

In conclusion, *KRAS* mutations were more common in females and smokers, but are not associated with COPD-status in NSCLC patients. *EGFR* mutations are more common in females and non-smoking NSCLC patients.

## Supporting Information

S1 Table Patients data used for statistical analysis. (XLSX)
